# The Management of Shunt Dysfunction Caused by Stent Deviation During Transjugular Intrahepatic Portosystemic Shunt (TIPS) Surgery for Esophageal and Gastric Varices Bleeding

**DOI:** 10.5152/tjg.2024.24243

**Published:** 2024-11-01

**Authors:** Jin-Mao Chen, Xiang-Long Li, Guo-Jie Zhang, Ning-Shen, Yu-Dong Wei, Feng Chen, Yan-Yan Cui

**Affiliations:** 1 Interventional Radiology, Heping Hospital Affiliated to Changzhi Medical College, Shanxi, China; 2 Department of Invasive Technology, Heping Hospital Affiliated to Changzhi Medical College, Shanxi, China; 3 Operating Centre, Heping Hospital Affiliated to Changzhi Medical College, Shanxi, China; 4 Neurology, Heping Hospital Affiliated to Changzhi Medical College, Shanxi, China

Dear Editor,

The transjugular intrahepatic portosystemic shunt (TIPS) is a sophisticated interventional technique that involves establishing a channel through the main branch of the portal vein via jugular access and inserting a stent to achieve effective portal shunting.^1^ However, the occurrence of stent restenosis following TIPS remains a significant obstacle to achieving optimal mid- and long-term therapeutic outcomes.

The occurrence of early shunt stenosis, within 1 month after surgery, is primarily attributed to improper positioning of the stent and inadequate postoperative anticoagulation therapy. However, other factors, such as incorrect placement or migration of the stent after surgery, insufficient length of the stent, failure to cover all shunts, insufficient tension or dilation during balloon catheterization, and excessive angulation in liver parenchyma, can also contribute to this condition. Currently, the established therapeutic approaches for addressing shunt dysfunction resulting from shunt stenosis involve thrombolysis, re-balloon dilatation, and stent repositioning.^2^ This article presents a case study on the migration of a stent during a TIPS procedure aimed at managing its diagnosis and subsequent intervention.

The patient, a 60-year-old male, was admitted to the hospital due to a 10-day history of hematemesis and melena. Following relevant examinations, the patient received diagnoses including liver cirrhosis with bleeding esophageal and gastric varices. After an emergency evaluation, TIPS surgery was planned.

After administering local anesthesia, we proceeded to puncture both the right internal jugular vein and femoral artery. Then, a vascular sheath was inserted into the right hepatic vein. We selectively catheterized the splenic artery, and the resulting angiogram revealed significant splenomegaly along with tortuous splenic arteries that were approximately twice as wide as those found in the common hepatic artery (as shown in  Figure 1A  ). Furthermore, indirect portal venography using the splenic artery indicated non-expansive masses (as illustrated in  Figure 1B ).

The right branch of the portal vein was successfully punctured via RUPS-100 through the right hepatic vein. A guide wire was inserted into the distal end of the splenic vein. Angiography showed tortuous and thickened gastric coronary veins ( Figure 1C  ). The gastric coronary veins were blocked, and the angiography revealed no progression in the distal aspect (as shown in  Figure 1D ).

The guiding catheter was retracted to the right hepatic vein, and a 6 mm × 60 mm balloon was inserted along the guidewire. Subsequently, the balloon was withdrawn, and a stent was successfully deployed. The catheter was then selectively introduced into the proper hepatic artery, where angiography revealed well-formed branches (as shown in  Figure 1E ).

The patient presented with complaints of upper abdominal pain, nausea, and vomiting. Physical examination revealed abdominal distention. Positive shifting dullness was observed during the examination. The color Doppler ultrasound examination of the portal vein revealed the presence of a stent positioned between the portal vein and inferior vena cava, indicating potential thrombosis (as shown in  Figure 2A  ). The subsequent treatment option considered was stent thrombus aspiration and balloon dilation.

The patient underwent a second procedure 5 days after the initial TIPS surgery. Fluoroscopy revealed an “angle break” at the junction of the TIPS stent cover and bare stent (as shown in  Figure 1F  ). With guidance from the guide wire, the catheter was selectively advanced into the main portal vein. Transcatheter angiography demonstrated that no contrast material passed through the TIPS stent. Selective catheterization of the superior mesenteric vein showed that when supported by the catheter, it straightened out the bending angle of the TIPS stent (as shown in  Figure 1G  ). Re-angiography confirmed the restoration of blood flow in the TIPS stent.

The surgical plan was modified as follows: a new bare stent was inserted within the original TIPS stent, and the misalignment between the original stent cover and the bare stent was rectified by the supportive action of the new stent. The hepatic venous return fell within the normal range (as shown in  Figure 1H ).

The portal vein color Doppler ultrasound revealed normal stent placement and unobstructed blood flow in the portal vein (as shown in  Figure 2B ).

Based on the timing of occurrence, TIPS shunt restenosis can be categorized into 2 groups: (1) Early stent stenosis is primarily caused by thrombotic occlusion and (2) mid- and long-term restenosis predominantly contributes to TIPS shunt failure. Following TIPS placement, various factors stimulate excessive hyperplasia of pseudointimal tissue, leading to shunt stenosis and occlusion. The key to preventing portal vein thrombosis lies in proper stent placement during the TIPS procedure and maintaining stent patency postoperatively.^3^

The ultrasound examination is the preferred method for assessing stent patency following TIPS.^4^ The primary ultrasound criterion for identifying abnormal TIPS is a blood flow velocity reduction exceeding 50%.

Currently, commonly employed interventional treatment methods for managing shunt dysfunction include angioplasty, re-stent implantation, and parallel shunt establishment. The specific measures for stent occlusion include: (1) Performing transjugular shunt recanalization, wherein a balloon catheter is introduced into the stenotic shunt through the jugular vein for dilation, achieving recanalization in most cases; (2) Considering relining the stent for patients with poor efficacy of simple balloon catheter dilation to achieve better long-term patency; (3) If the shunt cannot be reopened, re-puncture and establishment of a parallel shunt can be done.^5^ During the second operation, no thrombosis was observed but there were signs of a “broken angle” at the junction area between the covered stent and bare stent due to blood vessel tortuosity during initial placement. The shape of the blood vessel affected the stent resulting in an excessive angle. The treatment plan was adjusted to insert a bare stent within the original one, allowing dual stents to provide support.

Ensuring prompt identification and management of stent restenosis following the TIPS procedure is crucial for maintaining uninterrupted shunt patency and preventing the recurrence of portal hypertension.

## Figures and Tables

**Figure 1.**
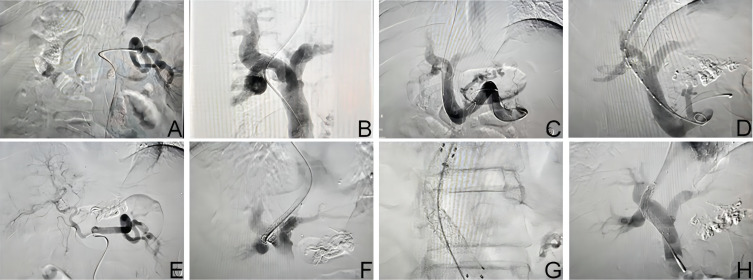
The splenic artery was tortuous on angiography. Fig. B: The main portal vein and the right branch showed significant thickening. Fig C: The coronary veins of the stomach exhibited tortuosity and hypertrophy. Fig. D: No stasis was observed in the distal gastric coronary vein following embolization. Fig. E: The stent was subsequently released after precise positioning. Fig. F: The break angle at the junction between the TIPS stent cover and bare stent was observed. Fig. G: The TIPS stent was straightened with the assistance of a catheter for support. Fig. H: The placement of the bare stent was successfully performed, ensuring unobstructed blood flow.

**Figure 2.**
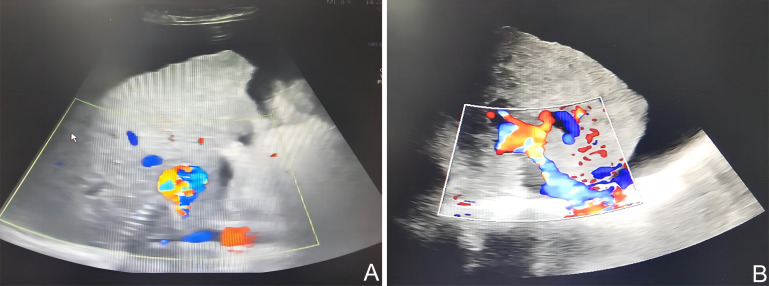
The presence of portal vein thrombosis was evaluated using color Doppler ultrasound.

**Figure 3.**
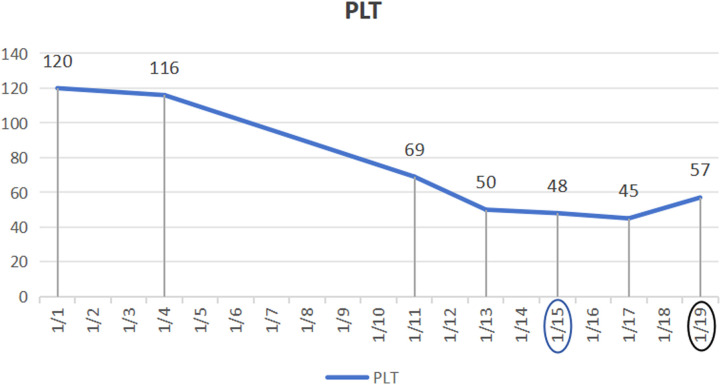
The trend of changes in platelet indices.

**Figure 4.**
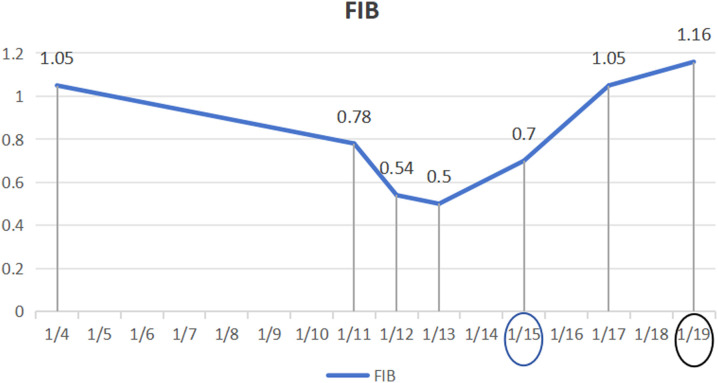
The evolving pattern of the fibrinogen measurement index.

## Data Availability

The data that support the findings of this study are available on request from the corresponding author..
